# Impact of Short-Term Topical Steroid Therapy on Selective Laser Trabeculoplasty Efficacy

**DOI:** 10.3390/jcm10184249

**Published:** 2021-09-19

**Authors:** Tomaž Gračner

**Affiliations:** 1 Faculty of Medicine, University of Maribor, 2000 Maribor, Slovenia; tomaz.gracner@ukc-mb.si; Tel.: +386-40-522765; Fax: +386-23-312393; 2 Department of Ophthalmology, University Clinical Centre Maribor, 2000 Maribor, Slovenia

**Keywords:** selective laser trabeculoplasty, topical steroid therapy, primary open-angle glaucoma, intraocular pressure

## Abstract

Background: To evaluate whether short-term use of topical steroid therapy affected the efficacy of selective laser trabeculoplasty (SLT) for primary open-glaucoma (POAG). Methods: 25 eyes of 25 patients, who used a drop of dexamethasone 0.1% 4 times a day for 7 days as post-laser therapy, formed the Steroid SLT group and 24 eyes of 24 patients, where no topical steroids or nonsteroidal anti-inflammatory agents as post-laser therapy were used, formed the No-steroid SLT group. Success was defined as an intraocular pressure (IOP) lowering exceeding 20% of pretreatment IOP. Results: The mean follow-up time was 21.24 months for the Steroid SLT group and 20.25 months for the No-steroid SLT group (*p* = 0.990). No significant difference was found between the two groups for mean pretreatment IOP (22.20 mmHg vs. 22.33 mmHg), and for mean IOP reductions during whole follow-up period. At all follow-up visits, the mean IOP reductions were smaller in the Steroid SLT group than in the No-steroid SLT group. At all follow-up visits, the mean percent IOP reduction was smaller in the Steroid SLT group than in the No-steroid SLT group, and such a difference was significant at 12 months (25.4% vs. 29.6%, *p* = 0.047) and 24 months (25.3% vs. 29.7%, *p* = 0.024). According to the Kaplan–Meier survival analysis, the 24-month success rate was 84% in the Steroid SLT group and 79.2% in the No-steroid SLT group, with no differences between the groups (*p* = 0.675). Conclusion: Short-term use of topical steroid therapy had no impact on the efficacy of SLT for POAG.

## 1. Introduction

Selective laser trabeculoplasty (SLT), used since 1998 when the first successful protocol was described, has become an established method for lowering the intraocular pressure (IOP) in the treatment of open-angle glaucoma (OAG) and ocular hypertension (OH) [1,2,3,4,5]. Multiple prospective or retrospective studies clear demonstrated the safety and efficacy of SLT in reducing the IOP in eyes with OAG or OH [6,7,8,9,10,11,12,13,14,15,16,17]. Therefore, topical medical treatment and SLT are stated as initial, first line treatment options, and SLT is also an adjunctive treatment option in the treatment for OAG or OH in the latest 5th edition of *Terminology and Guidelines for Glaucoma* from the European Glaucoma Society [18].

Anti-inflammatory topical medication four times a day for 7 days after SLT treatment is described in many studies, although there is little evidence to support this [1,6,7,8,9,10,11,12,13,14]. Symptomatic or asymptomatic anterior chamber inflammation after SLT may occur, but usually resolves without treatment [1,5,19,20,21,22,23]. Treatment with topical steroids or nonsteroidal anti-inflammatory agents after SLT in most reports has not shown to cause a significant reduction in inflammation or improved efficacy, but it still remains a controversy in clinical practice [20,21,22,23,24,25].

This retrospective chart review evaluates whether short-term use of topical steroid therapy affected the efficacy of SLT for primary open-glaucoma (POAG) patients.

## 2. Materials and Methods

The patients selected for this retrospective chart review were recruited from the glaucoma unit of the Department of Ophthalmology, University Clinical Centre Maribor, Slovenia. All the eyes of the patients had POAG with uncontrolled IOP (>18 mmHg) on uppermost tolerated topical antiglaucoma medication and were treated with 180 degrees SLT. In the study we included consecutive patients, between January and December 2004, who used a drop of dexamethasone 0.1% four times a day for 7 days as post-laser therapy and they formed the Steroid SLT group. In the study we also included consecutive patients, between January and December 2014, where no topical steroids or nonsteroidal anti-inflammatory agents as post-laser therapy were used and they formed the No-steroid SLT group. We included patients of both genders, older than 50 years. Data such as age, sex, past and present ocular medication and ocular history were recorded. Exclusion criterion included a history of previous ocular surgery within 6 months, any previous glaucoma surgery, eye trauma, glaucoma laser therapy or uveitis to the study eye and any other form of glaucoma aside from POAG, such as normal-pressure glaucoma (NTG), pseudoexfoliative glaucoma (PXFG), or ocular hypertension (OH). Hazards, advantages, and substitutes of SLT treatment for POAG were explained to every patient and informed consent was obtained. Thus 43 eyes of 25 patients (18 bilateral) formed the Steroid SLT group and 41 eyes of 24 patients (17 bilateral) formed the No-steroid SLT group. There was just one eye per patient incorporated in every bilateral case in the study. The selection of eyes was random using a random numbers table, where the right eye was combined with even numbers and the left eye with odd numbers. The data of best corrected visual acuity, results of slit lamp examination, ophthalmoscopy, automated static perimetry (Swedish Interactive Threshold Algorithm [SITA] standard 30-2 program of the Humphrey Field Analyzer), and gonioscopy were collected. Trabecular meshwork pigmentation was graded according to a standard scale (graded from 0 to 4+ where 0 = no pigment and 4+ = dense homogeneous pigment). The IOP was measured with a Goldmann applanation tonometer. The baseline IOP presented the mean of three times measured preoperative IOPs in the 3 weeks prior to SLT treatment. One hour prior to SLT treatment IOP was measured and one drop of 0.5% apraclonidine was applied in the treated eye. The trabecular meshwork of every eye was treated with 50 adjacent but not overlapping spots in the inferior 180 degrees with a 532 nm frequency doubled Q-switched Nd:YAG laser (Selecta 7000; Coherent, Palo Alto, CA, USA). The same laser was used for all the procedures done in the year 2004 and also in the year 2014. The pulse duration was 3 ns with a single pulse and the spot size was 400 microns. The SLT treatment started using energy of 0.8 mJ, which was increased or decreased until only intermittent cavitation bubbles formation appeared. After SLT treatment, a drop of 0.5% apraclonidine and 0.1% dexamethasone were applied in the treated eye. All SLTs were performed by the same glaucoma specialist (G.T.) All the eyes underwent a slit lamp examination and applanation tonometry 1 h post-laser to assess the anterior chamber reaction and IOP spikes. Patients were evaluated 1, 3, 6, 12, 18, and 24 months after treatment. A failure was defined as any eye with IOP lowering less than 20% from baseline IOP 1 month post-laser. Hypotensive antiglaucoma medical therapy was not modified during study period. When any eye required either an alteration of hypotensive medical therapy, and thus failed to respond to SLT, that eye was excluded from further analysis at that point. Independent sample t tests were used in statistical analyses of comparing the groups. Significant *p* values were considered to be less than 0.05. All tests were performed two-tailed. Because of the variability in length of follow-up among patients, Kaplan–Maier life-table (survival) analysis was used to estimate the success rates for the groups. The two survival curves (success rates) were compared using the log-rank test. Statistical analysis was carried out with IBM SPSS Statistics version 22.0 for Windows.

## 3. Results

In the Steroid SLT group were 25 eyes of 25 patients, and in the No-steroid SLT group were 24 eyes of 24 patients. The baseline characteristics including number of patients, number of eyes, age, sex, vertical Cup/Disc ratio, mean deviation, number of hypotensive medications, best corrected visual acuity, trabecular meshwork pigmentation, and mean baseline IOP of the Steroid SLT group and the No-steroid SLT group are listed in Table 1. The mean pretreatment IOP in the Steroid SLT group was 22.20 mmHg (SD 2.5) and 22.33 mmHg (SD 2.6) in the No-steroid SLT group (*p* = 0.856). The differences between those baseline characteristics were statistically not significant, except the difference between mean energy used for each spot (*p* < 0.001) and total energy used (*p* < 0.001), which were higher in the No-steroid SLT group.

Treatment with SLT was conducted in all eyes with adjacent 50 spots in the inferior 180 degrees of the trabecular meshwork. The mean energy used for each spot was in the Steroid SLT group 0.76 mJ (SD 0.2) and 1.25 mJ (SD 0.1) in the No-steroid SLT group; the difference was statistically significant (*p* < 0.001). The total energy used was in the Steroid SLT group 37.63 mJ (SD 10.1) and 70.96 mJ (SD 13.8) in the No-steroid SLT group; the difference was statistically significant (*p* < 0.001).

Ophthalmic Nd:YAG laser is a solid-state laser and is pumped by a pulsed flashlamp. The lifetime of the Nd:YAG laser can last for 10 or more years. The limiting component, the one that needs to be replaced occasionally, is the flashlamp. Over the working years of the Nd:YAG laser, the function of the pulsed flashlamp slowly diminishes, so to achieve the desired laser effect, the energy of the laser spot must be increased. In our study, the same laser was used for all the procedures done in the year 2004 and also in the year 2014. The treatment protocol of the trabecular meshwork of every eye in our study was the same, the energy of the laser spot was set at the level of the appearance of intermittent cavitation formation. This explains the significant difference between mean energy used for each spot and total energy used, which were higher in the No-steroid SLT group treated in the year 2014.

Hypotensive antiglaucoma medical therapy in both groups was not modified during whole study period.

The mean follow-up time was for the Steroid SLT group 21.24 (SD 7.0) months and for the No-steroid SLT group 20.25 (SD 7.6) months; the difference was statistically not significant (*p* = 0.990).

The mean IOPs, mean IOP reduction and mean percent IOP reduction from baseline IOP 1, 3, 6, 12, 18 and 24 months after treatment for the Steroid SLT group and the No-steroid SLT group are listed in Table 2. The differences in the mean IOPs and the mean IOP reductions at different time intervals following SLT between the two groups were statistically not significant (*p* > 0.05). At all follow-up visits, the mean IOP reduction was smaller in the Steroid SLT group than in the No-steroid SLT group.

At all follow-up visits, the mean percent IOP reduction was smaller in the Steroid SLT group than in the No-steroid SLT group, and such a difference was statistically significant at 12 months (25.4% (SD 4.8) vs. 29.6% (SD 8.2) (*p* = 0.047)), and 24 months (25.3% (SD 5.5) vs. 29.7% (SD 6.5) (*p* = 0.024)).

**Figure 1:**
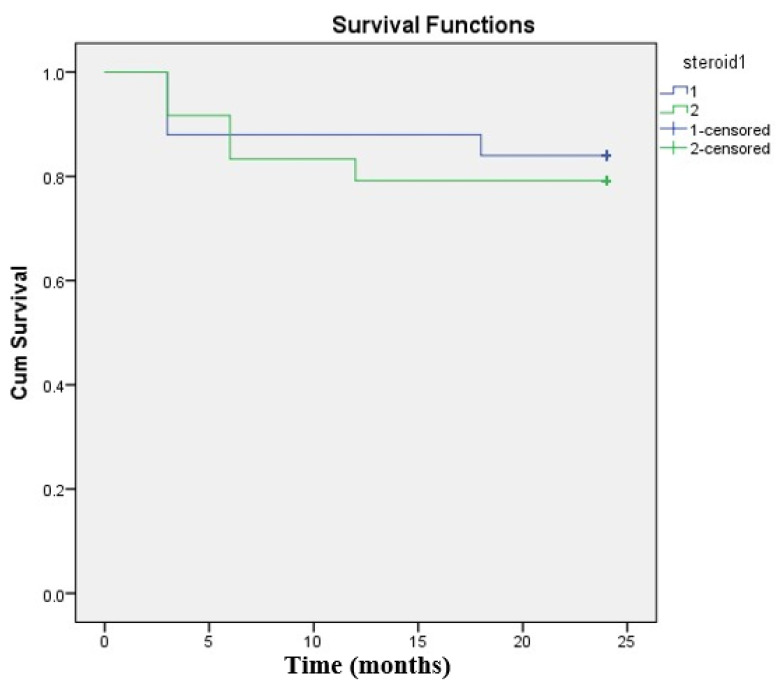
Kaplan–Meier survival analysis for the Steroid SLT group (group1) and the No-steroid SLT group (group 2).

In the Steroid SLT group, 4 eyes failed to respond to SLT (3 eyes after 3 months and 1 eye after 18 months), and in the No-steroid SLT group 5 eyes failed to respond to SLT (2 eyes after 3 months, 2 eyes after 6 months and 1 eye after 12 months). The success rate after 24 months determined from Kaplan–Meier life-table (survival) analysis was 84% in the Steroid SLT group and 79.2% in the No-steroid SLT group. By the comparison of the two survival curves (success rates) with the log-rank test there was no statistically significant difference (*p* = 0.675) between the groups (Figure 1).

After SLT there was no significant anterior segment inflammation or a transient increase in IOP in any of the treated eyes detected. No patient suffered any pain or inconvenience whilst they were treated.

## 4. Discussion

SLT is a laser procedure that selectively targets pigmented trabecular meshwork cells without causing thermal damage or collateral damage to nonpigmented cells or structures [26,27,28,29,30]. A 532 nm Q-switched frequency doubled Nd:YAG laser with a fixed spot size of 400 microns and pulse duration of 3 nanoseconds is used for SLT. The power range for treatment using currently available laser platforms is 0.3 to 2.0 mJ. The exact mechanism of action of reducing the IOP in this procedure is not completely understood and is likely multifactorial. The demonstrable clinical efficacy of SLT, despite the absence of coagulation of the trabecular meshwork suggests that laser trabeculoplasty works on the cellular level either through migration and phagocytosis of trabecular meshwork debris by the macrophages, or by stimulation of formation of healthy trabecular tissue, which may enhance the outflow properties of the trabecular meshwork [31]. Alvorado et al. has observed a five to eight fold increase in the number of monocytes and macrophages present in the trabecular meshwork of monkey eyes treated with SLT as compared with untreated controls [32]. They theorized that injury to the pigmented trabecular meshwork cells after SLT results in the release of factors and chemoatractants, which recruit monocytes which are activated and transformed into macrophages upon interacting with the injured tissues. These macrophages then engulf and clear the pigment granules from the trabecular meshwork tissues and exit the eye to return to the circulation via the Schlemm’s canal [32]. The biological theory of SLT action implies a cascade of events (interleukins-1, tumor necrosis factor-a, matrix metalloproteinases, recruitment and increase in number of macrophages) triggered by the laser that causes the remodeling of the extracellular matrix in the non-treated areas of TM, so this remodeling presumably decreases the outflow resistance and hence decreases IOP [33,34,35,36,37,38,39]. All these events have been postulated to play a role in the IOP lowering effect of SLT. Short-term anti-inflammatory topical medication is commonly prescribed post SLT to ease early inflammation. Because of the proposed mechanism of action of SLT including production of pro-inflammatory cytokines, the potential counterproductive nature of prescribing topical anti-inflammatory medication has been considered.

Realini et al., in their prospective, randomized study, evaluated 25 POAG patients following bilateral 360° SLT, who in one randomly selected eye (25 eyes) used prednisolone acetate 1% 4 times daily for 1 week; the other eye (25 eyes) did not receive any anti-inflammatory treatment [20]. No significant difference in IOP-lowering effect was found between groups after a follow-up of 3 months [20].

Jinapriya et al., in their randomized, double-masked, placebo-controlled clinical trial, evaluated 125 patients with POAG or PXFG, 46 eyes treated with prednisolone acetate 1%, 41 eyes with ketorolac tromethamine 0.5%, or 38 eyes with placebo 4 times per day for 5 days after 180° SLT [21]. No significant difference in IOP-lowering effect was observed among the groups up to 1 year after treatment [21].

De Keyser et al., in their prospective, randomized clinical trial, evaluated 66 patients with either POAG, NTG, or OH following bilateral 360° SLT, who in one eye used indomethacin 0.1% (35 eyes) or dexamethasone 0.1% (31 eyes) three times daily for 1 week; the other eye did not receive any anti-inflammatory treatment [23]. No significant difference in anterior chamber reaction, conjunctival redness, reported pain, or IOP-lowering effect between groups after all time points with a follow-up of 6 months was found [23].

Groth et al., in their randomized, double-masked, placebo-controlled clinical trial, evaluated 96 eyes with either POAG (72 eyes), PXFG (4 eyes), or OHT (20 eyes) following 180° SLT (34 eyes), 270° SLT (11 eyes), or 360° SLT (50 eyes) [24]. Of these, 28 eyes (20 POAG, 8 OHT) were treated with ketorolac 0.5%, 37 eyes (28 POAG, 3 PXFG, 6 OHT) with prednisolone 1%, or 31 eyes (24 POAG, 1 PXFG, 6 OHT) with placebo four times per day for 5 days after SLT [24]. No significant difference in IOP decrease among groups was observed at week 6 of follow-up; both the nonsteroidal anti-inflammatory drug and steroid groups showed a significantly greater decrease in IOP at week 12 of follow-up compared with the placebo group [24].

Thrane et al., in their randomized, placebo-controlled clinical trial, evaluated 39 eyes with either POAG (10 eyes), PXFG (8 eyes), OHT (8 eyes), or NTG (13 eyes) following 360° SLT [25]. Of these, 19 eyes (6 POAG, 4 PXFG, 3 OHT, 6 NTG) were treated with diclofenac 0.1%, and 20 eyes with placebo (4 POAG, 4 PXFG, 5 OHT, 7 NTG) 4 times per day for 5 days after SLT [25]. No significant difference in IOP-lowering effect was observed among the groups after a follow-up of 6 months [25].

In the first SLT surgical technique protocol described by Latina et al. the use of short-term topical steroid therapy fourtimes a day for 7 days as postoperative management after SLT was postulated [1]. Most of the published studies until 2006 followed the prescribed operative SLT protocol, including our reports [1,6,7,8,9,10,11,12,13,14,19]. The mechanism by which SLT lowers IOP was investigated by many studies [26,27,28,29,30,31,32,33,34,35,36,37]. According to these findings, the short-term topical anti-inflammatory therapy after SLT became questionable. In many later studies the use of the short-term topical anti-inflammatory therapy after SLT was given on, including ours [15,16,17]. Therefore, we decided to evaluate whether short-term use of topical steroid therapy after SLT affected the efficacy of SLT in a retrospective chart review, in which we included POAG patients treated 2004, who were using short-term topical steroid therapy after SLT and compared the results with POAG patients treated 2014, who received no topical steroids or nonsteroidal anti-inflammatory agents after SLT.

In our retrospective chart review we evaluated 49 eyes with POAG following 180° SLT. Of these, 25 eyes were treated with dexamethasone 0.1% four times per day for 7 days after SLT and 24 eyes did not receive any topical steroids or nonsteroidal anti-inflammatory agents after SLT. No significant difference was found between the two groups in IOP reductions during whole follow-up period, with a follow-up of 24 months. Moreover, no significant difference was found between the two groups in success rate after a follow-up of 24 months. Short-term use of topical steroid therapy in our study had no impact on the efficacy of SLT for POAG.

Because of differences in age, gender, ocular history, type of antiglaucoma medications, amount of glaucomatous optic neuropathy, SLT treatment parameters, amount of included eyes, follow-up time, assessment of IOP reduction, study design, assessment, and statistical analysis of the results, a comparison of the mentioned studies is difficult and its possibility limited. The results of our study, where we evaluated whether short-term use of topical steroid therapy affected the efficacy of SLT for POAG, are similar to those previously reported [20,21,22,23,24]. The follow-up in our study was 24 months, therefore longer than in reported studies, where the follow-up was 3 to 12 months [20,21,22,23,24]. As in other published studies, our study with longer follow-up found the use of short-term topical anti-inflammatory medication after SLT for POAG makes no difference [20,21,22,23,24]. We also conclude that the IOP reduction is not influenced by the use of short-term topical anti-inflammatory medication after SLT.

No consensus statement exists regarding the postoperative management of patients after SLT. Larger additional long-term outcome studies that include a variety of glaucoma subtypes and different surgical techniques may be necessary to further investigate this issue.

## Figures and Tables

**Table 1:** Baseline characteristics—all patients.

	Steroid SLT Group	No-Steroid SLT Group	*p*
Patients (No)	25	24	0.911
Eyes (No)	25	24	0.911
Mean age (years) (SD)	70.44 (8.6)	67.00 (12.0)	0.255
Sex: Male	12	12	
Female	13	12	0.855
Vertical Cup/Disc Ratio (mean) (SD)	0.75 (0.3)	0.85 (0.2)	0.173
Mean Deviation (mean) (dB) (SD)	−9.22 (1.3)	−10.33 (1.4)	0.754
Hypotensive medication (mean) (SD)	2.4 (0.7)	2.3 (0.5)	0.875
Best corrected visual acuity (SD)	0.78 (0.3)	0.71 (0.4)	0.413
Trabecular meshwork pigmentation (mean) (SD)	1.92 (0.8)	2.17 (0.8)	0.265
Mean energy/spot (mJ) (SD)	0.76 (0.2)	1.25 (0.1)	<0.001
Total energy (mJ) (SD)	37.63 (19.1)	70.96 (13.8)	<0.001
Mean baseline IOP (mmHg) (SD)	22.20 (2.5)	22.33 (2.6)	0.856

No—Number; (SD)—Standard deviation; *p*—Independent sample *t* test; SLT—selective laser trabeculoplasty.

**Table 2:** Mean IOP, mean IOP reduction, and mean percent IOP reduction from baseline IOP at different time intervals following SLT–all patients.

Follow-Up Time	Eyes (No)	Eyes (No)	Mean IOP (mm Hg) (SD)	Mean IOP (mm Hg) (SD)		Mean IOP Reduction (mm Hg) (SD)	Mean IOP Reduction (mm Hg) (SD)		Mean % IOP Reduction (mm Hg) (SD)	Mean % IOP Reduction (mm Hg) (SD)	
	Steroid SLT Group	No-Steroid SLT Group	Steroid SLT Group	No-Steroid SLT Group	*p*	Steroid SLT Group	No-Steroid SLT Group	*p*	Steroid SLT Group	No-Steroid SLT Group	*p*
BASELINE	25	24	22.20 (2.5)	22.33 (2.6)	0.856	-	-	-	-	-	-
1month	25	24	16.72 (2.7)	17.10 (3.8)	0.699	5.48 (1.9)	5.49 (3.8)	0.976	25.1 (7.9)	26.6 (10.2)	0.547
3 months	22	22	16.14 (2.3)	15.90 (2.5)	0.705	6.09 (1.8)	6.45 (1.8)	0.520	27.4 (7.2)	28.9 (6.5)	0.459
6 months	22	20	16.00 (2.3)	15.60 (2.2)	0.570	6.23 (2.2)	6.55 (2.2)	0.633	27.8 (8.1)	29.5 (7.5)	0.505
12 months	22	19	16.64 (2.0)	15.26 (2.6)	0.053	5.59 (1.5)	6.68 (2.6)	0.102	25.4 (4.8)	29.6 (8.2)	0.047
18 months	21	19	16.05 (1.9)	15.05 (1.6)	0.080	6.19 (1.9)	6.95 (2.4)	0.274	27.7 (6.3)	30.8 (7.3)	0.154
24 months	21	19	16.62 (2.1)	15.32 (1.4)	0.051	5.62 (1.5)	6.58 (2.4)	0.137	25.3 (5.5)	29.7 (6.5)	0.024

No—Number; (SD)—Standard deviation; *p*—Independent sample *t* test; IOP—intraocular pressure.

## Data Availability

The data presented in this study are available on request from the corresponding author on reasonable request.
